# Assessing the use of constructs from the consolidated framework for implementation research in U.S. rural cancer screening promotion programs: a systematic search and scoping review

**DOI:** 10.1186/s12913-022-08976-2

**Published:** 2023-01-18

**Authors:** Jennifer L. Moss, Kelsey C. Stoltzfus, Madyson L. Popalis, William A. Calo, Jennifer L. Kraschnewski

**Affiliations:** 1 Penn State College of Medicine, Hershey, PA USA; 2 Department of Family and Community Medicine, Department of Public Health Sciences, Penn State College of Medicine, The Pennsylvania State University, 90 Hope Drive, #2120E, MC A172, P.O. Box 855, Hershey, PA 17033 USA

**Keywords:** Consolidated Framework for Implementation Research (CFIR), Cancer screening, Rural, Non-metropolitan, Program planning, Implementation science

## Abstract

**Background:**

Cancer screening is suboptimal in rural areas, and interventions are needed to improve uptake. The Consolidated Framework for Implementation Research (CFIR) is a widely-used implementation science framework to optimize planning and delivery of evidence-based interventions, which may be particularly useful for screening promotion in rural areas. We examined the discussion of CFIR-defined domains and constructs in programs to improve cancer screening in rural areas.

**Methods:**

We conducted a systematic search of research databases (e.g., Medline, CINAHL) to identify studies (published through November 2022) of cancer screening promotion programs delivered in rural areas in the United States. We identified 166 records, and 15 studies were included. Next, two reviewers used a standardized abstraction tool to conduct a critical scoping review of CFIR constructs in rural cancer screening promotion programs.

**Results:**

Each study reported at least some CFIR domains and constructs, but studies varied in how they were reported. Broadly, constructs from the domains of Process, Intervention, and Outer setting were commonly reported, but constructs from the domains of Inner setting and Individuals were less commonly reported. The most common constructs were planning (100% of studies reporting), followed by adaptability, cosmopolitanism, and reflecting and evaluating (86.7% for each). No studies reported tension for change, self-efficacy, or opinion leader.

**Conclusions:**

Leveraging CFIR in the planning and delivery of cancer screening promotion programs in rural areas can improve program implementation. Additional studies are needed to evaluate the impact of underutilized CFIR domains, i.e., Inner setting and Individuals, on cancer screening programs.

**Supplementary Information:**

The online version contains supplementary material available at 10.1186/s12913-022-08976-2.

Cancer incidence and mortality rates are generally higher in rural areas than in urban areas [1, 2]. Contributing to the elevated mortality rates is the less common uptake of routine screening tests for breast, cervical, and colorectal cancers in rural areas [3–7]; across cancer types, the prevalence of screening is as much as 10% lower in rural areas. Differences in cancer screening compound the elevated burden of select cancer risk factors in rural areas, e.g., higher rates of tobacco use [8] and lower levels of physical activity [9]. Certain barriers to cancer screening, such as travel distance, are more pertinent for screening among rural compared to urban populations [10–14]. As a result, interventions to promote cancer screening need to be responsive to the specific needs of rural populations, including their context and health systems [15]. Failure to develop or adapt interventions in this way could exacerbate urban/rural differences in cancer screening and outcomes.

The Consolidated Framework for Implementation Research (CFIR) [16, 17] outlines implementation domains and constructs that influence the success of interventions, particularly when implementing these interventions in new contexts. The five CFIR domains are *Intervention* (i.e., the components and characteristics of the program itself), *Inner setting* (i.e., immediate context in which the intervention will be implemented), *Outer setting* (i.e., the larger social context surrounding the implementation setting), *Individuals* (i.e., those actors who implement the intervention), and *Process* (i.e., the anticipated process for achieving change); each domain includes 4–14 constructs and sub-constructs [16]. For example, the domain of Inner setting includes the constructs of structural characteristics, networks and communication, culture, implementation climate, and readiness for implementation. Targeting these domains and constructs could improve the quality of program implementation by identifying multilevel barriers and facilitators to evidence-based interventions, e.g., cancer screening promotion programs in rural settings. However, the extent to which rural cancer screening promotion programs assess and address constructs from implementation science, including those from CFIR, is unknown.

This study involves a systematic search and scoping review of published studies on rural cancer screening promotion programs in order to characterize how interventions considered barriers and facilitators to implementation that map on to CFIR constructs. Specifically, we evaluated whether programs (a) explicitly addressed these constructs, and (b) how the constructs informed program planning and delivery. These findings can illustrate the progress and gaps in application of implementation science to rural cancer screening promotion.

Although multiple implementations science frameworks and models exist [18], we chose to structure our work around CFIR because it is well-established with cancer control researchers and practitioners, and it has well-defined, distinct constructs relevant to our review. The findings will highlight the extent to which implementation science has been used in rural cancer screening promotion and highlight areas in which additional data are needed. More broadly, the findings will help inform research and quality improvement efforts for increasing cancer screening in rural areas.

## Methods

### Procedures for study identification

We used standardized procedures for conducting a systematic literature search and scoping review of studies published through November 2022 [19]. This approach involved (a) a comprehensive search of the published research literature and (b) a critical, narrative review and summary. The review focused specifically on the use of CFIR domains and constructs.

First, we developed a list of inclusion/exclusion criteria and search terms (Supplemental Table S1), which we refined with the assistance of a health science librarian. Briefly, eligible studies were those that reported on programs conducted in the United States with cancer screening as a primary study outcome. We included published protocol papers and outcomes papers, but excluded reviews, meta-analyses, and commentaries. All available literature was included, regardless of publication date; although some studies predated the initial publication of CFIR in 2009 [16], this framework described constructs that already existed in the field of implementation science. Search terms included constructs related to (a) evaluation of interventions (e.g., “process evaluation,” “evidence-based practice”), (b) rural settings (e.g., “rural,” “non-metropolitan”), and (c) cancer screening (e.g., “screen*,” “early detection of cancer”). Studies that self-identified their settings as rural were included; that is, we did not apply any additional restrictions on what qualified as a rural setting. To be eligible, studies had to intervene in at least one rural site, including studies with rural-only site(s) and studies with rural and non-rural sites. Then, we conducted a comprehensive search of the scientific literature archived in Medline, CINAHL, Scopus, Cochrane, Web of Science, and Embase.

**Fig. 1:**
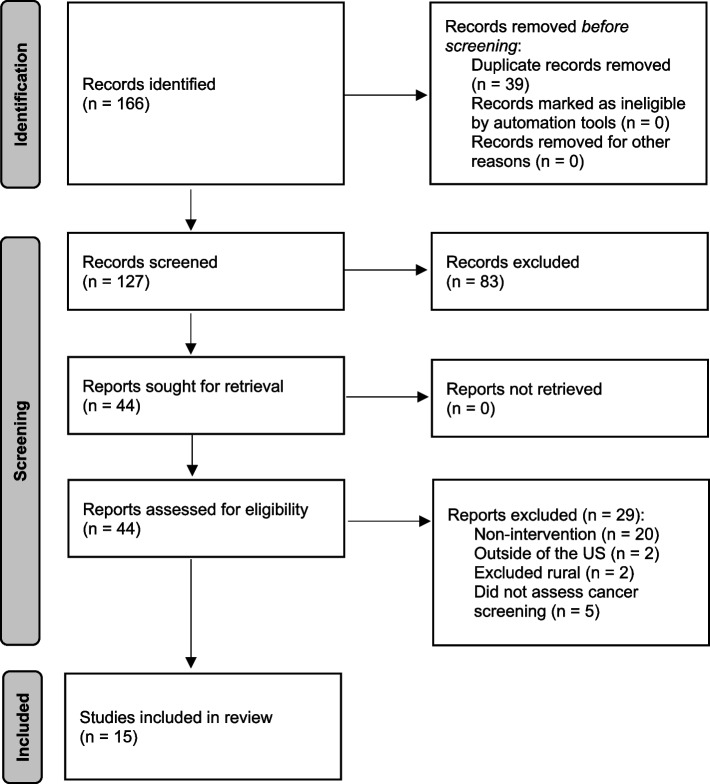
Results of a systematic search on the planning and delivery of studies evaluating rural cancer screening promotion programs in the United States, using the Preferred Reporting Items for Systematic Reviews and Meta-Analyses (PRISMA) flow diagram

We used the Preferred Reporting Items for Systematic Reviews and Meta-Analysis Extension for Scoping Reviews (PRISMA-ScR; https://www.prisma-statement.org/Extensions/ScopingReviews) guidelines to inform the search (Fig. 1). In the systematic search, we identified 166 records for review; 39 of these were duplicate publications that we removed. Two study team members (JLM and KCS) reviewed titles and abstracts for the remaining 127 records, achieving a 98% (125/127) agreement rate. A third team member (WAC) reviewed the titles and abstracts for the records in conflict to resolve the disagreements. Forty-four studies were included in the full-text review, assessed by two independent reviewers (JLM and KCS). Twenty-nine studies were excluded from further analysis, most often (20/29, or 69%) because they did not describe an intervention, e.g., they evaluated pre-existing cohort data or described theoretical/conceptual work on rural cancer screening. Ultimately, 15 studies describing rural cancer screening promotion programs were included in the critical review (Table 1) [20–34].

**Table 1:** Characteristics of included studies evaluating rural cancer screening promotion programs in the United States

First author	Year	Location	Target population	Program summary	Targeted cancer screening outcome
Viadro	1997	Five counties in eastern North Carolina	Women, age 50 + , African American	The program included assisting local health departments and community health centers in enhancing existing breast cancer screening services; reducing practical barriers to screening (e.g., high cost); and increasing awareness of breast cancer and screening among African American women	Breast: Mammography
Sharp	1998	North Carolina	Women, age 18 + , members of Eastern Band of Cherokee Indians and Lumbee Tribe	A lay health educator provided two in-home visits with an educational video, brochure, discussions, and tailored health information about cervical cancer detection and screening	Cervical: Pap smear
Cunningham	2000	Six counties in western North Carolina	Women, age 20 + , low income	The educational program at county health departments included media messages, direct education, telephone counseling, staff education, and small workshops	Skin: Clinical skin exam, skin self-exam Cervical: Pap smear Breast: Clinical breast exam, mammography, breast self-exam
Bencivenga	2008	One county in western Pennsylvania	Women, age 40 + , visiting a food pantry, eligible for breast cancer screening	Staff hung educational posters, developed a radio campaign, and stuffed promotional flyers in food bags for food pantry patrons. Women at the pantries were assessed for breast cancer screening eligibility, and staff provided navigation to schedule and identify financial support for mammography	Breast: Mammography
Teal	2012	Two counties in eastern North Carolina	Women, age 40 + , African American	The program relied on lay health advisors to conduct one-on-one and small group discussions with patients to schedule and identify financial support for mammography	Breast: Mammography
Norman	2013	Sixteen counties in eastern Colorado	Adults, age 40 +	"Boot camp" process to translate evidence-based prevention guidelines into lay messages and campaigns (one exemplar topic was colon cancer prevention). The boot camp process for colon cancer prevention involved 1 full-day retreat, 4 half-day retreats, and 8 half-hour phone calls. The lay campaign included newspaper stories about community members, a farm auction flyer, pocket-sized cards with locally-tailored photographs, community education sessions, and a travel mug with a colon cancer prevention message	Colorectal: Colonoscopy and other screening
Lee-Lin	2014	Two communities in Oregon	Women, age 40 + , eligible for breast cancer screening	The multicomponent, community-specific intervention included appointment setting, financial assistance, one-on-one client education, reminder letters from a clinician, breast health education DVD, and breast cancer screening continuing medical education to clinicians	Breast: Mammography
Breslau	2015	Underserved counties in six states (Alabama, Georgia, Kentucky, Missouri, South Carolina, and Tennessee)	Women, low income, eligible for breast or cervical cancer screening	State and community organizations worked with a federal network to choose, adapt, and implement evidence-based interventions to increase breast and cervical cancer screening. Specific interventions differed across sites	Breast: Mammography Cervical: Pap smear
Cardarelli	2017	Two communities in eastern Kentucky	Adults, age 55–77, eligible for lung cancer screening	The tailored community awareness campaign about lung cancer screening disseminated flyers, postcards, and radio and newspaper ads. In addition, the campaign included a website and provided education to clinicians	Lung: Low-dose CT
Gesthalter	2017	Three Veterans Administration clinics in three states	Not specified	Three VA clinics were classified as early adopters of lung cancer screening with low-dose CT. Specific interventions differed across sites	Lung: Low-dose CT
Katz	2017	Twelve counties in Appalachian Ohio	Adults, age 51–75	The media campaign disseminated information about colon cancer screening through billboards, posters, and newspaper articles	Colorectal: Any screening
Schlauderaff	2017	One rural health clinic in western Washington	Adult patients, age 50–75	The program included clinic-focused efforts: Additional staff education, standardizing protocols for clinicians to discuss and order screening, implementing a flag for screening in the electronic health record system, and improved systems for interprofessional communication. In addition, the program included patient-focused efforts: Mass mailing of stool tests, financial assistance, and outreach	Colorectal: Any screening
Woodall	2018	One city government workplace in Kentucky	Adult employees, age 18 +	The program nurse delivered educational sessions about colorectal cancer screening to city employees. The nurse also distributed free stool tests and communicated test results to participants, providing referral to primary care clinicians if needed	Colorectal: Stool testing
Tolma	2019	One tribal community in Oklahoma	Women, age 52–74, American Indian, eligible for breast cancer screening, with a scheduled clinic visit in the next month	The program targeted systems change in the clinic and community. Clinic interventions included training to clinicians to support patient-doctor communication on mammography, including a tailored mammography brochure, sending follow-up recommendation letters, developing a screening flowchart, and hanging an educational bulletin board poster at the clinic. Community interventions included discussion groups for eligible women and a younger female relative, as well as a small gift after mammography	Breast: Mammography
Elliott	2021	Thirty-four primary care clinics in four states	Patients, age 11–75, eligible for cancer screening or prevention services, with a clinic visit during the study period	Primary care clinics instituted a web-based clinical decision support tool or shared decision making tools, integrated into the electronic health record, to prompt clinicians to prioritize delivery of primary and secondary cancer prevention services to eligible patients	Composite measure of breast, cervical, and colorectal cancer screening (any modality)

*CT* Computed tomography

### Data extraction and synthesis

To organize data abstraction, we developed a review tool following best practices [35]. The tool included fields focused on bibliographic data; intervention structure (e.g., geographic scope, study design, target sample); intervention dose (e.g., number of visits); intervention mode (e.g., type of materials, language of delivery); and intervention effects, if available. The remainder of the tool was devoted to closed- and open-ended items to assess the 39 CFIR constructs and sub-constructs. The tool included definitions [16] of each construct that reviewers could reference while evaluating a study.

We abstracted data from the included studies to investigate the use of CFIR domains and constructs through two complementary approaches. First, we evaluated the presence or absence of CFIR constructs in each study, creating quantitative, binary indicators for each construct. Two reviewers (JLM and KCS) independently evaluated the presence of each CFIR construct for every study and then met to generate consensus. Importantly, studies may have referred to CFIR constructs explicitly or implicitly, so we determined that a construct was ‘present’ if the study included information that mapped on to the construct *definition/meaning* even if the study did not use the construct *label*.

We pilot-tested the data abstraction tool and process by independently reviewing one study and meeting to debrief and resolve questions. Revisions to the tool were made to clarify questions that arose during the pilot-testing process (e.g., emphasizing the difference between an opinion leader and a champion). The reviewers then independently reviewed ~ 5 studies at a time, meeting every 1–2 weeks to debrief and protect against ‘drift’ in data abstraction. The reviewers achieved an 81% overall agreement rate for the presence/absence of each CFIR construct (agreement by CFIR domain: Intervention: 77%; Inner setting: 89%; Outer setting: 77%; Individuals: 90%; Process: 80%). If necessary, disagreements were resolved through discussion with a third reviewer (WC).

Second, we extracted summary information about how studies described each reported construct in program development and/or implementation. We then summarized the prevalence of CFIR constructs and how they informed program development and/or implementation for the included studies of rural cancer screening promotion.

## Results

We reviewed a total of 15 studies published between 1997 and 2021 (Table 1). As a result of the inclusion criteria for our review, all studies described interventions with some prospective, longitudinal data collection. All programs targeted adults (18–75 +), but age eligibility varied across programs. Some programs focused recruitment on participants with certain characteristics, such as a specific race or ethnicity [20, 21, 24, 33] or low income [22, 23, 27]. Studies examined mammography for breast cancer [20, 22–24, 26, 33], Pap smear for cervical cancer [21, 22], low-dose computed tomography (CT) for lung cancer [28, 29], colorectal cancer screening tests (fecal immunochemical test (FIT) and colonoscopy) [30–32], or skin cancer checks [22]. On average, studies described 17 CFIR constructs (range: 10–26) (Table 2).

**Table 2:** Domains, constructs, and sub-constructs from the Consolidated Framework for Implementation Research [16] present in included studies evaluating rural cancer screening promotion programs in the United States

	**Study**															
	Viadro, 1997 [20]	Sharp, 1998 [21]	Cunningham, 2000 [22]	Bencivenga, 2008 [23]	Teal, 2012 [24]	Norman, 2013 [25]	Lee-Lin, 2014 [26]	Breslau, 2015 [27]	Cardarelli, 2017 [28]	Gesthalter, 2017 [29]	Katz, 2017 [30]	Schlauderaff, 2017 [31]	Woodall, 2018 [32]	Tolma, 2019 [33]	Elliott, 2021 [34]	Studies reporting, *n* (%)
***Intervention***																
Intervention source		X		X	X	X	X	X		X	X			X		9 (60%)
Evidence strength and quality	X			X	X	X	X			X					X	7 (47%)
Relative advantage				X	X	X	X	X			X					6 (40%)
Adaptability	X	X	X	X	X	X	X	X	X	X	X		X	X		13 (87%)
Trialability					X					X				X	X	4 (27%)
Complexity	X	X	X	X	X	X	X	X		X				X	X	11 (73%)
Design quality and packaging			X			X	X		X						X	5 (33%)
Cost	X	X	X	X			X		X	X	X	X	X		X	11 (73%)
***Outer setting***																
Patient needs and resources	X	X		X		X	X	X	X		X	X	X	X	X	12 (80%)
Cosmopolitanism		X	X	X	X	X	X	X	X	X	X	X	X	X		13 (87%)
Peer pressure							X								X	2 (13%)
External policy and incentives	X			X	X	X			X	X		X				7 (47%)
***Inner setting***																
Structural characteristics				X	X	X	X	X			X	X			X	8 (53%)
Networks and communications	X	X		X	X	X	X	X		X		X	X		X	11 (73%)
Culture		X			X			X								3 (20%)
Implementation climate					X			X							X	3 (20%)
Tension for change																0 (0%)
Compatibility	X	X	X			X	X	X		X		X		X	X	10 (67%)
Relative priority			X		X	X		X		X						5 (33%)
Organizational incentives and rewards						X								X		2 (13%)
Goals and feedback		X	X										X	X	X	5 (33%)
Learning climate		X	X		X	X	X									5 (33%)
Readiness for implementation					X				X					X		3 (20%)
Leadership engagement		X	X		X	X		X		X		X	X	X	X	10 (67%)
Available resources	X	X	X	X	X	X	X	X	X			X			X	11 (73%)
Access to knowledge and information		X	X	X		X	X	X	X			X			X	9 (60%)
***Individuals***																
Knowledge and beliefs about the intervention						X	X			X		X	X		X	6 (40%)
Self-efficacy																0 (0%)
Individual stage of change										X						1 (7%)
Individual identification with organization						X										1 (7%)
Other personal attributes		X			X		X									3 (20%)
***Process***																
Planning	X	X	X	X	X	X	X	X	X	X	X	X	X	X	X	15 (100%)
Engaging	X	X			X	X		X	X	X	X			X		9 (60%)
Opinion leader																0 (0%)
Formally-appointed internal implementation leaders	X		X		X	X				X		X			X	7 (47%)
Champions	X					X	X	X		X		X				6 (40%)
External change agents						X						X				2 (13%)
Executing	X	X	X	X			X	X	X	X	X		X		X	11 (73%)
Reflecting and evaluating	X	X	X	X	X	X	X	X		X		X	X	X	X	13 (87%)

### CFIR domain: intervention

Within the Intervention domain, the least commonly-discussed construct was *trialability* (27%), and the most commonly-discussed construct was *adaptability* (87%) (Table 2).

Two programs were developed from an external *intervention source*, such as the American Cancer Society [23, 27]. More programs were developed from an internal *intervention source* based on collaborations between community and academic partners [24–26, 29, 30] and through formative research such as focus groups and pilot interventions [21, 30]. To demonstrate *evidence strength and quality* of the program, studies reported on systematic reviews [26], expert advice and education [20, 25], and evidence-based research [23, 24, 29, 34]. Studies indicated the *relative advantage* of a given program in two ways: (a) emphasizing the importance of selecting evidence-based programs over other options, and (b) asserting that programs that were culturally-appropriate for the target population would be more effective at changing behavior [23–27, 30].

A majority of studies described the *adaptability* of a program by summarizing refinements to the program’s core components to ensure it would resonate with the target population, including cultural relevance [21, 28, 32, 33] and local knowledge [25, 26, 30]. Some studies discussed *adaptability* in relation to the ‘adaptable periphery,’ [16] such as program elements [22, 23, 27] and systems related to the program [20, 24, 29]. For example, Sharp et al. described their work to promote cervical cancer screening among American Indian women in rural North Carolina, which they adapted from a previous program targeted at African American women in urban North Carolina [21]. Before program implementation, they introduced adaptations to (a) make the approach more individualized rather than community-based, and (b) increase the relevance to rural, Southern, and American Indian culture. They made additional adaptations during implementation, as well, including changing protocols to allow for more flexible scheduling and revising the format of the individualized risk assessment results. Thus, reported adaptations were multilevel and ongoing throughout this and other studies.

*Trialability*, or testing the program in a smaller group within the target population, was infrequently discussed [24, 29, 33, 34]. In terms of *complexity* of program implementation, several studies described difficulties associated with perceptions about (and reality of) the burden of staff trainings and responsibilities [20, 21, 24, 33], additional workload [22, 23, 25, 27, 29, 34], and partners’ time and resource commitment [26].

*Design quality and packaging*, focusing on design elements, literacy level, and program messaging, were evaluated by focus group participants or community members [22, 25, 26, 28, 34]; usually, this feedback was solicited before implementation [22, 25, 28, 34], but other studies gathered this information after implementation [26]. A majority of studies discussed program *costs*, focusing on program implementation (such as staff training, travel, and management [20–23, 26, 29, 31, 34]) rather than specific program components (such as free cancer screening tests [30, 32] and advertisements [28]).

### CFIR domain: outer setting

Within the Outer setting domain, the least commonly-discussed construct was *peer pressure* (13%), and the most commonly-discussed construct was *cosmopolitanism* (87%) (Table 2).

In describing *patient needs and resources*, studies included (a) epidemiological data about cancer burden [21, 23, 25–28, 30–33], cancer risk factors, or risk factors for forgoing cancer screening [23, 26–28, 30, 31, 33, 34] or (b) qualitative or formative research about the specific needs of the community members [20, 21, 25–28, 33]. Most studies reported on this construct when describing the motivation for locating a program in a particular community, although CFIR suggests that *patient needs and resources* should be addressed in each stage of program development and implementation. Notably, none of these studies described resources, assets, or strengths; instead, they focused on the needs, deficits, and weaknesses experienced by patients in the local community.

*Cosmopolitanism*, or the extent to which program settings were networked with other, external organizations, was incredibly varied across studies in terms of number and types of organizations in the network. Studies reported collaborations that involved leaders of community organizations [21, 24, 27], public health programs/departments [22, 23, 25, 27, 29, 30], healthcare clinics or providers [23, 25, 28, 31, 32], and universities [24, 26, 28], among others [23, 26, 32, 33]. While most studies described partnerships with 1–5 external organizations, some studies were part of quite large networks; for example, Norman et al. [25] developed a colorectal cancer screening program through the High Plains Research Network, which is comprised of “16 community hospitals, 55 practices, 120 primary care clinicians, 20 nursing homes, several public health departments, and about 145,000 residents.”

*Peer pressure* [26, 34] and *external policy and incentives* [20, 23–25, 28, 29, 31] were discussed less frequently. Most often, *external policy* referred to federal policies that mandated funding for specific public health or clinical programs.

### CFIR domain: inner setting

Within the Inner setting domain, the least commonly-discussed construct was *tension for change* (0%), and the most commonly-discussed constructs were *networks and communications* and *available resources* (both 73%) (Table 2).

Descriptions of *structural characteristics* of the program settings highlighted their history [23, 26, 30, 31, 34], physical resources [25, 26], and staff support [24, 25, 27]. *Networks and communications* within program settings were often described in detail [20, 21, 23–27, 29, 31, 32, 34]. Many studies outlined the systematized communication among departments or team members for the duration of program implementation [21, 29, 31, 32, 34]; notably, these systems were almost invariably reported as facilitators of implementation without discussion of how challenges emerged or were addressed [27]. Reported elements of *culture* of a program setting focused on how a given program aligned with institutional priorities [27], particularly around serving the community [21, 24].

Reports of the *implementation climate* were relatively infrequent in the studies reviewed, except in descriptions of how team members worked together collaboratively in support of the intervention [24, 27, 34]. Program teams prepared for, and adapted during, implementation by working to maximize *compatibility* among the program and the clinic/organization systems [26, 27, 29, 31, 33, 34], implementation teams [20–22], and patient expectations of the program setting [21, 25, 33]. Many of these efforts focused on established pathways for integrating screening tests from a research study into the clinical records. Several studies reported low *relative priority* of focusing on a particular topic, i.e., that staff may have perceived other health issues as higher priorities than cancer screening [24, 27, 29], but none specified how they attempted to modify this perception. Notably, none of the studies described the *tension for change* within an organization (beyond describing the data indicating a need for change in the community; see CFIR domains: Outer setting).

The construct of *readiness for implementation* consists of “specific tangible and immediate indicators of organizational commitment to its decision to implement an intervention” [16], and it includes the sub-constructs of *leadership engagement*, *available resources*, and *access to knowledge and information*. *Leadership engagement* often involved increasing the commitment and involvement of leaders from the program setting by connecting them with a research team [22, 24, 27, 34] or local community leaders (e.g., elected officials, hospital administrators) [21, 25, 27, 29, 32, 33]. *Available resources* in program settings included tangible assets [21, 23–25, 28, 31, 34] (such as materials or physical space) and intangible resources [20, 21, 34] (such as training/education). Three studies reported on the lack of *available resources*: (a) Breslau et al. [27] reported that insufficient funds challenged implementation of cancer screening promotion; (b) Cunningham et al. [22] reported that, compared to a better-resourced health department, a health department with fewer resources was able to adopt program innovations more easily because staff did not have to integrate the new procedures with existing practices; and (c) Elliott et al. [34] reported that the COVID-19 pandemic reduced their capacity to recruit and intervene with patients.

### CFIR domain: individuals

Descriptions of the Individuals (that is, individuals within the program setting, not the participants in the research projects) were infrequent. Within this domain, the least commonly-discussed construct was *self-efficacy* (0%), and the most commonly-discussed construct was *knowledge and beliefs about the intervention* (40%) (Table 2).

*Knowledge and beliefs about the intervention* [25, 26, 29, 31, 32, 34] was primarily addressed through use of educational sessions and training [25, 31, 34], knowledge assessments [32], and providing research evidence to program staff in lay language [26]. One study documented staff ambivalence to changes in clinical practice due to skepticism about the program, but this was mitigated through additional peer-led educational sessions [29]. The role of *individual stage of change* was integrated into one program that leveraged cross-discipline, collaborative communication to move intervention staff through stages of change during program implementation [29].

*Individual identification with an organization* was discussed by one study that utilized a community advisory council in program implementation [25]; this approach allowed all participants to have the opportunity to contribute ideas and fostered an environment for success. Of the three studies that discussed *other personal attributes*, Teal et al. [24] described the knowledge gap between community and academic partners that may have impeded program implementation; Lee-Lin et al. [26] designed program materials that could be used by inexperienced yet capable health educators; and Sharp et al. [21] emphasized to community health workers that they needed to stay in compliance with their own medical care in order to effectively encourage community participants to engage with cancer screening.

### CFIR domain: process

Within the Process domain, the least commonly-discussed construct was *opinion leader* (0%), and the most commonly-discussed construct was *planning* (100%) (Table 2).

*Planning* was a crucial component in the development and reporting of all of the studies, involving formative (often qualitative) research [20, 22, 25, 26, 28, 32, 33], pilot studies [21, 27, 30], and time dedicated to training program staff (ranging from 9–12 months) [20, 23, 24, 29, 34]. Occasionally, *planning* included preplanned interim evaluation and adjustment throughout the program period [22, 31], e.g., several cycles of “plan, do, study, act” [31].

The sub-constructs within *engaging* outline four different roles for representatives from the implementation setting and other organizations (e.g., staff, volunteers, researchers) to be included in the implementation process [16]. Beyond individuals with a formal leadership role in the projects, none of the studies identified individuals as *opinion leaders*. However, *formally appointed internal implementation leaders* (primarily, staff embedded in the program setting [20, 22, 24, 25, 29, 31, 34]) and *champions* (primarily, staff with exceptionally high levels of motivation to go “above and beyond” their specified program responsibilities [20, 25–27, 29, 31]) were more common. For example, Lee-Lin et al. [26] reported that having a “passionate local health educator was a critical success factor” for their rural cancer screening promotion program.

Studies reported successes [20, 26, 30, 32], failures [21, 23, 27–29], and adaptations [21, 22, 26, 30, 34] in *executing* the program according to plan. Methods used to track *executing* the plan included extensive monitoring tools to ensure fidelity to the implementation plan [20–22, 29, 30, 34]. *Reflecting and evaluating* – that is, multidirectional, quantitative and qualitative communication about implementation efforts among team members – were described in great detail, leveraging multiple sources of information, numerous modes of communication, and creative methods for tracking implementation outcomes. Two examples that illustrate the breadth and depth of data collection for *reflecting and evaluating* are (a) Cunningham et al. [22], who described their routine communication, which was supplemented with presentations from the evaluation team to the program setting, weekly phone calls, quarterly in-person monitoring visits, and quarterly training workshops, and (b) Tolma et al. [33], who collected implementation data through activity logs, one-on-one interviews, phone calls, forms, checklists, surveys, and focus groups. Some studies reported gaps in their *reflecting and evaluating* [20, 26], noting that they did not have the information they needed to evaluate the implementation of specific program components.

## Discussion

Among 15 studies of cancer screening promotion programs delivered in rural U.S. areas, all described at least some elements of CFIR that the authors leveraged during the development or implementation of the program. Most commonly, these studies described constructs from the Process, Intervention, and Outer setting domains, including planning, adaptability, and cosmopolitanism. However, constructs from the Inner setting and Individuals domains were described less frequently. While the focus of implementation science frameworks, such as CFIR, is on improving the quality and success of program implementation, they may also improve the effectiveness of behavioral programs [36–38]. As such, it is possible that leveraging a framework such as CFIR when developing and implementing these rural cancer screening programs can increase this behavior and potentially improve cancer outcomes. Recent publications from authors at the U.S. National Cancer Institute and Centers for Disease Control and Prevention emphasize the role of implementation science in developing and delivering better interventions to improve cancer screening and reduce disparities, including guiding the development of programs that are culturally-tailored [39], community-based [39, 40], multilevel [39, 40], and scalable [39–41]. As a result of the ongoing COVID-19 pandemic, changes to health policy and healthcare delivery make it even more important to understand how to best deliver cancer screening promotion programs to communities and patients that need them most. Additional research is needed on how to improve the use of implementation science to promote cancer screening in rural areas, particularly in terms of optimizing programmatic elements such as internal dynamics (i.e., Inner setting) and staff/stakeholders (i.e., Individuals).

### The people in the programs: the role of networks in rural cancer screening promotion programs

Some studies emphasized that people and organizations in their networks played multifaceted roles in the planning and delivery of these programs, such that these roles spanned several CFIR domains, including Outer setting (e.g., *cosmopolitanism*), Inner setting (e.g., *networks and communication*), Individuals (e.g., *knowledge and beliefs*), and Process (e.g., *engaging*) domains. Engagement of community stakeholders is a critical component of effective implementation [27, 39, 40, 42, 43], although community-engaged research approaches are underutilized [44, 45]. Collaboration with community members and community organizations may be particularly important for rural cancer control given the tight relationships and small networks in rural communities [46]. Although half of the studies described internal implementation leaders and champions (i.e., roles internal to the study team), only two studies described external change agents, and none described opinion leaders (see engaging construct under Process domain).

External change agents often have training in a technical field germane to the program (e.g., paid consultants) [16]. External change agents may be hard for community programs to identify and pay, thereby reducing sustainability of cancer screening programs; as such, the contributions of external change agents (if leveraged) must be measured and reported to increase transparency.

Opinion leaders are “individuals in an organization who have formal or informal influence on the attitudes and beliefs of their colleagues with respect to implementing the intervention” [16] and may be experts or peers, e.g., tribal leaders, religious leaders, or other local community organization leaders. A review of randomized controlled trials found that leveraging opinion leaders can change the behaviors of healthcare professionals between -15% to + 72%, i.e., they can have positive of negative effects [47]. Understanding the most advantageous ways to involve opinion leaders is a pressing need for program implementation.

In addition, many programs reported collaborations with external organizations, some of which were quite large (see cosmopolitanism construct under Outer Setting domain); these relationships can be crucial for successful implementation [48]. Interestingly, communication among people and organizations during program implementation was always described positively in these studies (see networks and communications construct under Inner Setting domain), with little discussion of challenges and potential solutions. Approaches such as network analysis could provide more insight into the density and functionality of networks of healthcare and community-based organizations, particularly in rural areas where opportunities for collaboration may be constrained by geography [49, 50]. In addition, some authors have called for greater attention to communication theory in implementation science research [51, 52]; these paradigms could provide insight into how network nodes co-create priorities, information, and resources related to public health and cancer screening. An overarching approach to supporting these networks would be to employ greater team science [15] to ensure that team formation, launch, and maturation are all adequately supported throughout the program lifespan.

### Understudied domains and constructs in reviewed studies

Among the included studies, there was limited discussion of certain constructs within the Individuals or Inner setting domains. Two out of the three constructs that were not discussed by any study came from these domains: tension for change (domain: Inner setting) and self-efficacy (domain: Individuals).

Tension for change can be understood as stakeholders’ perceptions of a discrepancy between current practice and what (better) practice is possible [53]. This discrepancy can be driven by patients’ needs (e.g., unacceptably low levels of cancer screening) or by expectations from leadership [53–55]. If stakeholders do not feel that change is necessary or even possible, they will have low motivation to support the program, but if stakeholders view the status quo as intolerable, they are more likely to support the program [56, 57]. Gustafson et al. [58] suggest that interventionists will find tension for change difficult to impact, and time burden (and burnout) among healthcare staff can preclude their ability to engage meaningfully in program activities even if they do have tension for change [16, 59, 60]. However, tension for change may wax and wane organically over time [55], and if opinion leaders within an organization have high tension for change, this sentiment can spread throughout the network of stakeholders [58]. These issues highlight the interconnected constructs of tension for change and engagement; additional research is needed to understand the interactions among these variables in predicting program success.

In terms of self-efficacy, CFIR proposes that stakeholders’ self-efficacy for implementation influences their confidence in recognizing barriers and overcoming challenges, and makes them more likely to support the program, even as challenges arise [16, 61]. While decades of research have linked *participants’* self-efficacy to health behavior change [62], stakeholders’ self-efficacy is less well-studied. Approaches to improve self-efficacy could focus on simplifying or systematizing interventions or building capacity through training or technical assistance [61]. Efforts to provide training to stakeholders in rural areas can be challenged by time and travel concerns [63], but telementoring programs such as Project ECHO have been successful in improving the self-efficacy of rural healthcare providers to enact cancer prevention and control initiatives in their clinics [64, 65]. Future research should investigate how to train stakeholders in rural areas to implement cancer screening programs, and what impact this training has on intervention outcomes.

Several other constructs were discussed in less than 25% of studies: peer pressure (domain: Outer setting); culture, organizational incentives and rewards (domain: Inner setting); individual stage of change, individual identification with organization, other personal attributes (domain: Individuals); and external change agents (domain: Process). A recent systematic review concluded that behavioral health researchers should use flexible strategies for assessing Outer setting constructs, which are understudied [66]. Efforts to develop reliable and valid scales to assess a range of CFIR constructs specific to cancer control are underway; the availability of psychometrically-robust measures may stimulate measurement of these constructs in future studies [67, 68]. Actively measuring and monitoring constructs from these domains could improve intervention outcomes. Future studies implementing cancer screening programs in rural settings should better leverage aspects of the Individuals and Inner setting domains to improve intervention implementation and outcomes.

### Challenges to implementation of rural cancer screening promotion programs

In general, studies highlighted certain CFIR constructs as challenges to successful implementation of cancer screening promotion programs. Program complexity and cost (constructs from the Intervention domain) were commonly discussed, often hand-in-hand, because the time, effort, and resources needed to plan and deliver these programs were often high and potentially prohibitive outside of a grant-funded context. Other studies have highlighted the importance of these two constructs, which are barriers to long-term sustainability [69, 70]. Despite the *scientific* complexity that may motivate evidence-based cancer screening promotion programs, it is clear that the *practical* application of these programs must be as simple as possible to ensure widespread dissemination and sustainability, particularly for programs focused on low-resource communities and reducing health disparities [71].

Another challenge was relative priority (from the Inner setting domain), which is perhaps not surprising given the finite resources (tangible and human) available for public health and clinical practice. Gesthalter and colleagues reported their efforts to address knowledge and beliefs about their program among skeptical staff [29], emphasizing the importance of engaging both “hearts” (i.e., relative priority) and “minds” (i.e., knowledge/beliefs) of program implementers [72]. Yet challenges from program complexity and cost may make efforts to get buy-in from implementers infeasible. For example, the program implemented by Gesthalter and colleagues added educational sessions with program staff, but these sessions increase time and costs associated with program implementation. Efforts to efficiently engage implementers are a crucial next step for implementation science research focused on cancer screening promotion programs.

### Strengths and limitations

Strengths of this review include the focus on CFIR [16], a comprehensive framework commonly used in cancer-related implementation studies, with domains and constructs applicable to the broad scope of cancer screening promotion programs. We also reported on a largely understudied topic area, identifying key gaps in the collection and reporting of implementation measures in the context of rural cancer screening promotion. This information is necessary to support better implementation of programs in rural settings and help reduce urban/rural differences in cancer screening and outcomes. Another strength was the search in six major databases to capture relevant articles.

Due to large variability in study designs, reporting, and outcomes, a main limitation of this review was our inability to assess associations between program implementation and effect sizes. We did not assess study quality in this review, which also likely influences intervention effect sizes. Future studies should evaluate the quality of implementation, quality of intervention, and intervention outcomes. Although reported as a strength, our exclusive focus on CFIR constructs was also a limitation. Tabak and colleagues [18] have reported at least 61 framework and models for implementation science research. We may have excluded some studies reporting other implementation measures not present in CFIR or conceptualized in different ways. In addition, we identified some studies reporting on measures that predate CFIR; it is possible that some authors, pre-CFIR, did not use implementation science terminology or report on these measures even if they were collected. Overall, it is possible that studies reporting the development and implementation of rural cancer screening programs were excluded due to these issues, our search strategy, and/or delays in program implementation and reporting as a result of the COVID-19 pandemic. Recently, best practices for reporting implementation studies have been developed, and authors should adhere to such guidelines to the extent possible [73]. Urban/rural comparisons were not possible given the predominant focus on rural settings.

## Conclusions

In conclusion, implementation science, including CFIR, holds promise for improving the development and implementation of programs to promote cancer screening in rural communities yet has been largely underutilized in studies. Stronger integration of CFIR domains and constructs could improve intervention delivery and reduce urban/rural disparities in cancer outcomes [1, 2]. Published studies of rural cancer screening promotion programs often leveraged aspects of CFIR’s Process, Intervention, and Outer setting domains, highlighting the importance of supportive networks in the planning and delivery of these programs. These studies less frequently described constructs from the Individuals and Inner setting domains, which reveals avenues for additional research and evaluation. These findings can inform future programs to increase cancer screening in rural communities, particularly if they further examine the role of Individuals and Inner setting constructs that may influence implementation and effectiveness.

## Supplementary Information

**Additional file 1.**

## Data Availability

All data analyzed during this study are included in the article.

## Abbreviations

CFIR: Consolidated Framework for Implementation Research
CT: Computed tomography
FIT: Fecal immunochemical test
PRISMA: Preferred Reporting Items for Systematic Reviews and Meta-Analysis
