# Supplementary material for: Assessing the use of constructs from the consolidated framework for implementation research in U.S. rural cancer screening promotion programs: a systematic search and scoping review
Source: BMC Health Serv Res. 2023 Jan 18;23:48. doi: 10.1186/s12913-022-08976-2 (PMC9846667; doi:10.1186/s12913-022-08976-2)
Supplement: Supplementary file 1 — Additional file 1. [file 12913_2022_8976_MOESM1_ESM.docx]

**Supplemental Table S1.** Systematic search strategy and study inclusion and exclusion criteria for studies evaluating rural cancer screening promotion programs in the United States.

| **Systematic search strategy** | |
| --- | --- |
| ***Topic*** | ***Search terms*** |
| Implementation science  Rural populations  Screening  Cancer | (((Diffusion of Innovation[MeSH Terms]) OR (((((((process evaluation[Title/Abstract]) OR dissemination research[Title/Abstract]) OR Implementation Science[Title/Abstract]) OR Implementation research[Title/Abstract]) OR Implementation science[MeSH Terms]) OR Evidence-Based Practice[MeSH Terms]) OR Translational Medical Research[MeSH Terms]))) AND  ((((rural[Title/Abstract]) OR ("Rural Population"[MeSH Terms] OR "Rural Nursing"[ MeSH Terms] OR "Rural Health"[ MeSH Terms] OR "Rural Health Services"[ MeSH Terms] OR "Hospitals, Rural"[ MeSH Terms]))) OR ((((((non-urban[Title/Abstract]) OR nonurban[Title/Abstract]) OR non-metropolitan[Title/Abstract]) OR frontier[Title/Abstract]) OR remote[Title/Abstract]))) AND  ((((((Secondary Prevention[MeSH Terms]) OR Mass Screening[MeSH Terms]) OR Early Detection of Cancer[MeSH Terms]) OR Diagnosis[MeSH Terms]) OR Diagnosis[MeSH Subheading]) OR screen*[Title/Abstract])  Limit Cancer Subject search |

| **Study inclusion and exclusion criteria** | |
| --- | --- |
| ***Inclusion criteria*** | ***Exclusion criteria*** |
| Data collection takes place in US | Data collection takes place only in urban location |
| Assesses cancer screening behaviors | Non-peer reviewed publication  (e.g., opinion paper) |
| Describes design, implementation, or evaluation of an intervention or program | Non-original or empirical research  (e.g., commentary, systematic review, theoretical paper) |
| Publication is printed in English |  |
| Publication reporting results is peer-reviewed |  |
